# Physical Activity on Telomere Length as a Biomarker for Aging: A Systematic Review

**DOI:** 10.1186/s40798-022-00503-1

**Published:** 2022-09-04

**Authors:** Marlies Schellnegger, Alvin C. Lin, Niels Hammer, Lars-Peter Kamolz

**Affiliations:** 1 COREMED – Cooperative Centre for Regenerative Medicine, JOANNEUM RESEARCH Forschungsgesellschaft mbH, Neue Stiftingtalstrasse 2, 8010 Graz, Austria; 2 Division of Plastic, Aesthetic and Reconstructive Surgery, Department of Surgery, Medical University of Graz, Auenbruggerplatz 29, 8036 Graz, Austria; 3 Division of Macroscopic and Microscopic Anatomy, Gottfried Schatz Research Center for Cell Signaling, Metabolism and Aging, Medical University of Graz, Harrachgasse 21, 8010 Graz, Austria

**Keywords:** Physical activity, Exercise, Telomere length, Telomerase, Aging

## Abstract

**Background:**

Overall life expectancy continues to rise, approaching 80 years of age in several developed countries. However, healthy life expectancy lags far behind, which has, in turn, contributed to increasing costs in healthcare. One way to improve health and attenuate the socio-economic impact of an aging population is to increase overall fitness through physical activity. Telomere attrition or shortening is a well-known molecular marker in aging. As such, several studies have focused on whether exercise influences health and aging through telomere biology. This systematic review examines the recent literature on the effect of physical activity on telomere length (TL) and/or telomerase activity as molecular markers of aging.

**Methods:**

A focused search was performed in the databases PubMed and Web of Science for retrieving relevant articles over the past ten years. The search contained the following keywords: exercise, sport, physical activity, fitness, sedentary, physical inactivity, telomere, telomere length, t/s ratio, and telomerase. PRISMA guidelines for systematic reviews were observed.

**Results:**

A total of 43 articles were identified and categorized into randomized controlled trials (RCT), observational or interventional studies. RCTs (*n* = 8) showed inconsistent findings of increased TL length with physical activity in, e.g. obese, post-menopausal women. In comparison with a predominantly sedentary lifestyle, observational studies (*n* = 27) showed significantly longer TL with exercise of moderate to vigorous intensity; however, there was no consensus on the duration and type of physical activity and training modality. Interventional studies (*n* = 8) also showed similar findings of significantly longer TL prior to exercise intervention; however, these studies had smaller numbers of enrolled participants (mostly of high-performance athletes), and the physical activities covered a range of exercise intensities and duration. Amongst the selected studies, aerobic training of moderate to vigorous intensity is most prevalent. For telomere biology analysis, TL was determined mainly from leukocytes using qPCR. In some cases, especially in RCT and interventional studies, different sample types such as saliva, sperm, and muscle biopsies were analyzed; different leukocyte cell types and potential genetic markers in regulating telomere biology were also investigated.

**Conclusions:**

Taken together, physical activity with regular aerobic training of moderate to vigorous intensity appears to help preserve TL. However, the optimal intensity, duration of physical activity, as well as type of exercise still need to be further elucidated. Along with TL or telomerase activity, participants’ fitness level, the type of physical activity, and training modality should be assessed at different time points in future studies, with the plan for long-term follow-up. Reducing the amount of sedentary behavior may have a positive effect of preserving and increasing TL. Further molecular characterization of telomere biology in different cell types and tissues is required in order to draw definitive causal conclusions on how physical activity affects TL and aging.

**Key Points**

1. As life-expectancy increases, lifestyle choices like exercise take on increasing importance in healthy aging. Telomere attrition is a molecular marker of aging. Thus, physical activity may influence the aging process through telomere biology, namely TL and telomerase activity.
2. The amount of reduction in sedentary behavior appears to have a positive effect of preserving and increasing TL. The current level of physical fitness seems to have a more significant impact than the history of previous exercise on TL. Telomere dynamics are tissue- and cell-specific and are also dependent upon proliferative activity; as such, grasping the molecular mechanisms induced by exercise remains a challenge.
3. Detailed information should be included in future studies (e.g., participants’ characteristics, level of fitness, type of exercise training modality, telomere analysis) in order to achieve greater study homogeneity and draw causal conclusions on the effects of exercise on telomere dynamics and the aging process.

## Introduction

With advancements in healthcare and improvements in living standards, human life expectancy is now predicted to be above 80 years of age in industrialized countries [1, 2]. Subsequently, the proportion of the elderly population has steadily increased; and by 2050, approximately a quarter of the world’s population will be over 65 years in age [3]. With a growing and aging population, increased costs in health care delivery have also transpired amongst other socio-economic challenges [4]. Unfortunately, healthy life expectancy lags far behind overall life expectancy [5], which implies a more extended period of morbidity [6]. To help curtail this financial impact and improve overall health, primary prevention strategies that incorporate lifestyle choices of a heathy diet and regular exercise have been promoted [7–9]. Not surprisingly, the cosmetic industry continues to profit, with the search of anti-aging and rejuvenation products attracting great attention.

Aging is an inherent and complex biological process, with several studies furthering our understanding at the molecular level [10, 11]. Lopez-Otin et al. [12] describe the main hallmarks of aging with respect to the following underlying mechanisms: genomic instability, loss of proteostasis, epigenetic alterations, mitochondrial dysfunction, cellular senescence, stem cell exhaustion, altered intercellular communication, deregulated nutrient-sensing, and telomere attrition. Amongst these hallmarks, telomere attrition and the preservation of telomere length (TL) have attracted much attention as a molecular marker of biological age [13]. Capping the arms of each chromosome, telomeres are long repeated nucleotide sequences whose primary function is to protect the integrity of genomic DNA from degradation, thereby maintaining genomic stability throughout the cell cycle [14]. With each cell division, TL progressively shortens by approximately 50–100 base pairs (bp) [15]. As TL decreases over time, telomeres become too short for the cell to divide any further, leading to cellular senescence [16]. This relationship of aging with decreasing TL has been confirmed by several studies [17–19]. Telomerase, an enzyme containing the catalytic unit protein reverse transcriptase, is considered the primary driver for replicating telomeric regions. Telomerase activity in combination with TL help to reflect the cell’s proliferation potential [20].

Although TL and its attrition can be highly variable amongst individuals with possible sex-specific differences [17], TL remains stable from childhood to early adulthood and diminishes in late adulthood [16, 21]. Critically short telomeres are associated with chromosomal degradation, end-to-end fusion, and deficient recombination, all of which promote the process of aging and age-related pathologies [22, 23]. Furthermore, telomere attrition is also associated with several chronic diseases and pathological conditions such as diabetes, dyslipidemia, cardiovascular disease, cancer, and during psychological stress [21, 24–28]. As such, TL is increasingly recognized as a clinical tool in gauging the risk for age-related diseases [29]. Some epidemiologic studies have even underpinned an increased risk of premature mortality with TL shortening [30].

Whether age-associated decline can be counteracted by enhancing TL integrity or preserving TL through primary prevention measures remains to be elucidated. However, increasing data support that negative lifestyle risk factors such as smoking and obesity are associated with a deleterious effect on TL; and living an active lifestyle has a beneficial effect on preserving TL [31–33], suggesting the effects of anti-aging of physical activity at the cellular level [34]. Physical activity is long-considered to play a crucial role in health and aging by reducing the risk of developing several chronic conditions [35]. Some age-related diseases associated with shorter TL (e.g. diabetes mellitus, hypertension, or cardiovascular disease) are suggested to be prevented, managed, or even regressed by regular exercise [36–40].

The specific molecular mechanisms and potential counteracting measures of biological aging are highly complex. Aging, as defined by hallmark cellular processes of senescence, fibrosis, inflammation, and stem cell depletion in the presence of functional p53 [41], has been shown to be inversely proportional to TL at the cellular level. Furthermore, primary preventive measures of healthy lifestyle choices such as regular physical exercise increase healthy life expectancy. Also, the epidemiological evidence suggests a strong relationship of preserving TL with physical activity [42]. However, the impact of physical activity on aging (with TL as a molecular marker of aging) is not fully understood, as evidenced by 3 recent yet different systematic reviews. Valente et al. [43] pooled together a total of 7418 participants from 30 retrieved articles and reported that, with very-low level of certainty, physically active individuals have longer telomeres and stated that likely this effect was overestimated. Aguiar et al. [44] examined 11 retrieved studies on master athletes (which included their own study) and found that master athletes had longer telomeres in comparison with age-matched controls. Song et al. [45] searched for RCTs only and found inconclusive findings amongst their seven retrieved RCT studies, which included predominantly female participants and those diagnosed with cancer (e.g. breast cancer).

The possible impact of physical activity on TL and aging continues to be a topic of interest in sports medicine research and beyond; however, there lacks a consensus of the literature on whether the type of physical activity (or inactivity) could possibly account for these discrepancies in telomere biology and mechanisms that regulate TL. Therefore, this systematic review aims to examine the current literature on the impact of the level and type of physical activity on TL as a molecular marker of aging.

## Methods

A systematic review of the literature was conducted in keeping with PRISMA guidelines. The review protocol is registered on PROSPERO (record number: CRD42021252217).

### Search Strategy

The online databases PubMed and Web of Science were used to scan the available literature. The literature search was performed in May 2021 by two independent reviewers to identify the articles of interest based on the defined inclusion criteria. Any disagreements were solved by consensus, or a third-party reviewer was consulted when necessary. As a further measure to include relevant papers, references of included studies and retained reviews were also screened. In 2015, Munstock et al. [46] performed a complete search of the literature, and found only one RCT in 2014 for their review. As such, articles over a focused timeframe of 2011 to 2021 were included and analyzed.

The literature search was performed using a combination of the following search terms: “telomere” OR “telomeres” OR “telomerase” OR “telomere length” OR “t/s ratio” AND "physical activity" OR "physical exercise" OR “exercise” OR “motor activity” OR “locomotor activity” OR “active lifestyle” OR “inactive lifestyle” OR "physical inactive" OR sedentary OR sport OR “aerobic exercise” OR “training” OR “endurance training” OR “resistance training” OR “strength training” OR “isometric exercise” OR “isometric exercises” OR “interval training” OR “high intensity training” OR "fitness”.

Titles or abstracts of the identified studies were examined, and duplicates were removed using the reference software *Mendeley* (Elsevier B.V., Amsterdam, NL). Furthermore, the full text of the manuscript was examined if the title or abstract did not provide sufficient information. All studies that met the inclusion selection criteria were selected for full analysis and review.

### Selection Criteria

Articles were included if the following inclusion criteria were met:

1. The article assesses the impact of physical activity on telomere biology (TL and/or telomerase activity).
2. The research was limited to articles published in English or German from 2011 to 2021 and to articles involving a human testing population.
3. Full-text manuscripts of original studies (randomized controlled trial or RCT, observational, or interventional) that compared the participants’ characteristics (e.g. age, level of physical fitness), telomerase biology measurements (e.g. relative or absolute TL and/or telomerase activity), and physical activity (e.g. type, intensity, duration) were included.

Abstracts only, reviews, letters, responses, case series, case studies and duplicated articles were excluded. Studies that assessed the effect of holistic lifestyle interventions but did not include physical activity as one of the interventions were excluded. To achieve great study homogeneity, studies with participants who were under 18 years of age or with terminal illness were also excluded.

The following variables were defined as crucial data, with the goal to extract such information, if applicable, for each of the selected studies: study type, sample size, age, population, study protocol name, year or study timeframe, the type of tissue sample collected for telomere analysis, method of telomere analysis, type of physical activity (with description of its frequency, duration and intensity), physical fitness of participants, statistically significant results, and time of follow-up. All statistically significant results were screened for appropriate grouping, stratification, and corrections for potential bias, e.g. age-matched, risk factors, different scales or assessment parameters of physical fitness.

## Results

**Fig. 1:**
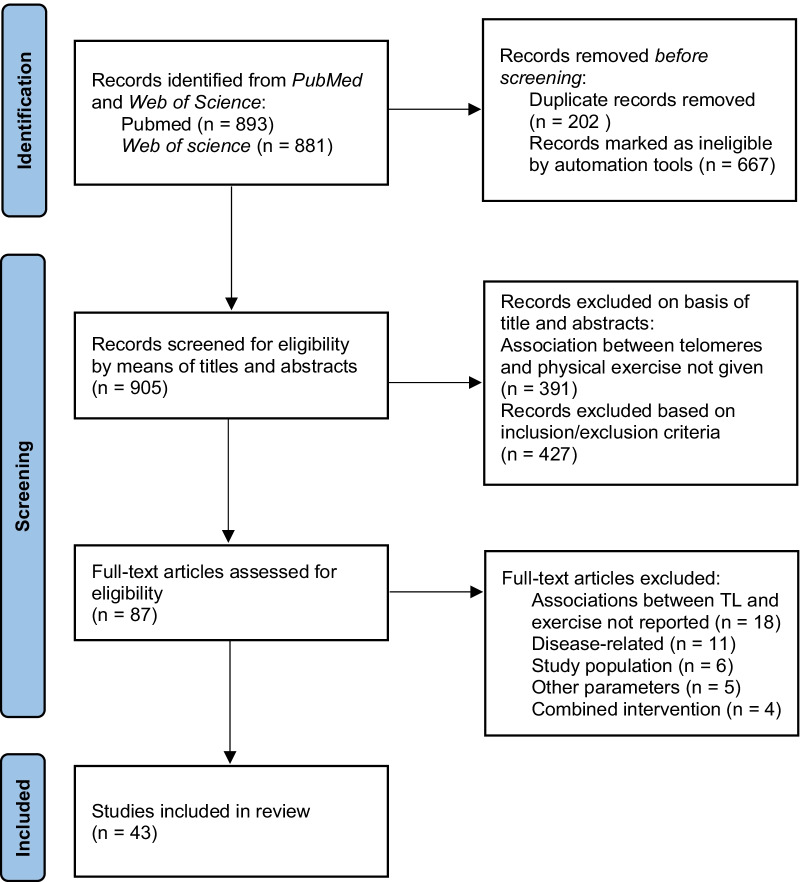
Flow chart of the systematic review process (PRISMA)

A flow chart summarizing the algorithm and the quantitative results of the search procedure is shown in Fig. 1. The initial literature search yielded a total of 1,774 studies. After removing duplicates, 905 remaining titles and abstracts were screened and analyzed based on the inclusion criteria described. Of the 905 records, 391 articles were excluded since these studies did not address telomere biology in association with exercise or vice versa. A further 427 records were excluded since they did not meet other inclusion criteria (e.g. human study population, original research, publication date, etc.). A total of 87 articles remained for full-text screening to further evaluate eligibility. Of these 87 articles, 44 articles were then excluded: eighteen studies did not assess any associations between parameters of telomere biology and exercise; eleven studies evaluated the effects of exercise on TL in relation to severe diseases (e.g. cancer); six articles focused on a study population that did not meet the inclusion criteria of age (e.g. children) or only in vitro experiments were conducted; five articles did not investigate one of the defined parameters; and four studies examined a combined intervention of several different lifestyle changes on telomere biology; however physical activity was not corrected for potential bias nor treated as independent variable. Taken together, the final number of articles included for this systematic review was 43.

For each of the selected articles, the study type was determined, and the article was then assigned into one of three groups: RCT (8/43), interventional (8/43), and observational (27/43) studies. A summary of the most important characteristics and findings of the selected studies are presented in Tables 1, 2, and 3. Several studies (33/43) investigated significant positive association between TL and physical activity. The most frequently utilized tissue- and cell-type for TL measurement were leukocytes (27/43), and qPCR was the most common method to evaluate changes in TL (33/43). A small number of studies (5/43) highlighted the acute effect of a single exercise bout.

**Table 1:** The effects of physical activity on telomere biology: *RCT studies*

References	Subjects	Telomere analysis: cell type	Measurement method	Physical activity	Main findings
Mason et al. [53]	439 women, postmenopausal, overweight or obese (50–75 years) *n* = 105 diet *n* = 106 aerobic exercise *n* = 108 diet + aerobic exercise *n* = 79 sedentary	TL: Leukocytes	qPCR	Aerobic PA at 225 min/week (5 sessions/week, 45 min/session) for 12 months; intensity of training reached at 70–85% of HR_max_, moderate to vigorous exercise	No significant difference in TL detected in any of the intervention group versus sedentary controls
Sjögren et al. [118]	*n* = 49 men and women randomly selected from previous RCT on PA invention versus sedentary lifestyle (68 years, 14 men)	TL: Whole blood	qPCR	PA of moderate- and low-intensity with pedometer tracking steps at 210 min/week exercise (30 min/d) for 6 months	↑ Significant association of increased TL with reduced sedentary/sitting time; No significant difference in TL with steps per day or increased exercise duration
Tosevska et al. [87]	105 men and women, elderly, sedentary lifestyle *n* = 23–30 RT exercise (82 ± 6 years) *n* = 21–23 RT exercise + protein supplement (81 ± 8 years) *n* = 20–26 sedentary group with cognitive training (84 ± 5 years)	TL: Whole blood	qPCR	RT at 2 sessions/week (elastic bands, chairs, body weight exercises), with 1 set of 15 repetitions for 4 weeks then progressive increase of 2 sets light exercises to 2 sets heavy exercises, for 6 months	No significant difference in TL in any of the groups
Friedenreich et al. [52]	212 women, postmenopausal, heathy, physically inactive *n* = 99 exercise (60.4 years) *n* = 113 sedentary (60.0 years)	TL: Leukocytes	qPCR	Aerobic PA at 225 min/week (5 sessions/week, 45 min/session) for 12 months; intensity of training reached at 70–80% of HR reserve	Increase in TL in the exercise group but not statistically significant due to inadequate sample size at follow-up
Puterman et al. [55]	68 men and women, caregivers *n* = 34 exercise (59.3 ± 5.7 years, 4 men) *n* = 34 sedentary (63.3 ± 6.4 years, 9 men)	TL: Leukocytes Telomerase: PBMC	TL: qPCR Telomerase: ddPCR	Aerobic PA at 60 min/week of lower moderate intensity 40% of HR reserve for 9 weeks; then 120–150 min/week of upper moderate intensity for 15 weeks	↑ Significant increase in rTL in the exercise group versus sedentary controls No significant difference in telomerase activity between groups
Eigendorf et al. [97]	291 women, sedentary lifestyle *n* = 146 ET exercise (53 ± 5 years) *n* = 145 sedentary (53 ± 5 years)	TL: PBMC	qPCR	ET at 210 min/week (> 3 sessions/week) for 6 months; intensity of training reached at individualized HR	↑ Significant increase in TL in the ET exercise group versus sedentary controls No effect on TL with aerobic exercise; only subjects of lowest aerobic fitness showed significant increase in TL
Werner et al. [57]	124 men and women, healthy, sedentary lifestyle *n* = 34 RT (48.1 ± 7.5 years, 14 men) *n* = 29 IT (48.4 ± 6.5 years, 10 men) *n* = 26 AET (49.5 ± 7.0 years, 9 men) *n* = 35 sedentary (50.2 ± 7.4 years, 12 men)	TL: MNCs, Leukocytes Telomerase: MNC	FACS, FISH, qPCR	PA training at 135 min/week (3 sessions/week, 45 min/session) for 6 months IT: 4 × 4 method, high-intensity AET: intensity at 60% heart rate RT: 8 machine-based exercises, 20 repetitions/exercise TL and Telomerase: at 0 and 6 months	↑ Significant increase in rTL and telomerase activity with AET after 6 months ↑ Significant increase in rTL with IT after 6 months No significant increase in TL or telomerase activity within RT or sedentary group after 6 months
Hooshmand et al. [58]	30 men, elderly, healthy, any level of regular physical activity *n* = 15 RT exercise (66.33 ± 3.35 years) *n* = 15 sedentary (66.13 ± 3.04 years)	Telomerase: Blood serum	ELISA	RT at 3 sessions/week (15 repetitions/exercise) for 12 weeks; intensity reached at 60-s with 30-s of rest, starting at 60% of repetition maximum with 5% increment in repetitions	↑ Significant increase in telomerase activity in the RT exercise group versus sedentary controls

**Table 2:** The effects of physical activity on telomere biology: *interventional studies*

References	Subjects	Cell type	Measurement method	Exercise intervention	Main results
Laye et al. [107]	8 men and 1 woman, endurance athletes (44 ± 2 years, 7 male)	TL, Telomerase (hTERT, hTERC) and shelterin complex proteins (e.g. TRF2): PBMC and skeletal muscle	qPCR	Endurance athletes: completed ≥ 1 ultra-marathon of ≥ 60 km PA: daily marathon distances, total 183 miles over 7 days TL & telomerase: blood samples and muscle biopsy pre- (2 wks before) and post- (22-24 h after) competition	No significant difference in rTL or telomerase activity with ultra-endurance PA (telomerase activity not detectable in skeletal muscle) Significant increase in mRNA expression of shelterin complex proteins in PBMCs Higher TRF2 found in skeletal muscle versus PBMCs
Borghini et al. [47]	62 men and women *n* = 20 endurance athletes (45.4 ± 9.2 years) *n* = 42 sedentary controls (45.9 ± 9.5 years)	TL: Saliva	qPCR	Endurance athletes: history of 59.4 km/week ultra-trail running for 13.15 years, ages grouped into tertiles PA: 330 km trail with incline of 24,000 m, duration of < 150 h TL: samples at baseline (1–2 d before race), intermediate point (148 km), and race completion (< 1 h) Controls: healthy, inactive, no competitive sports history	↓ Significantly reduced rTL in group of endurance athletes with PA (at intermediate point and at race completion) versus baseline ↑ Significantly longer TL in endurance athlete group (pre-PA) versus controls, largest difference of TL in older (≥ 53 years) group (pre-PA) versus age-matched controls
Diman et al. [131]	10 men, healthy, moderately active (20 ± 0.6 years) *n* = 5 low-intensity group *n* = 5 high-intensity group	TERRA: Skeletal muscle	qRT-PCR	PA: 45 min cycling, with target of 50% VO_2_ peak (low-intensity) and 75% VO_2_ peak (high-intensity) of endurance exercise TERRA: biopsies before, immediately after, and 2.5 h after PA	↑ Significant increase of TERRA levels immediately after and 2.5 h after PA versus before PA, for in both high- and low-intensity groups
Cluckey et al. [127]	19 men and women, healthy *n* = 11 young group (22 ± 2 years) *n* = 8 older group (60 ± 2 years)	Telomerase (hTERT and TRF2): PBMC	qRT-PCR	PA: 30 min cycling, varied workloads, with target of 90% VO_2_ max (high-intensity) TL: blood samples pre-PA, and at 30, 60, and 90 min post-PA	↑ Significant increase in relative hTERT levels with high-intensity PA versus pre-PA (greater increase in young group) Highest post-PA hTERT levels at 60 min for the older group and at 90-min for younger group ↑ Significantly greater increase in hTERT and TRF2 expression in men versus women, independent of age
Gagnon et al. [98]	25 men and women *n* = 15 expedition group (23.5 ± 7.4 years) *n* = 10 controls (31.1 ± 10.8 years)	TL: Leukocytes	qPCR	PA: 260 km canoeing expedition (6–9 h/d) low to moderate intensity, for 14 days TL: blood pre- (24 h before) and post- (3 h after) expedition	No significant change in TL between the expedition and control groups
Denham et al. [167]	34 men, healthy (31 ± 10 years)	TL: sperm	qPCR	Cardiopulmonary exercise test to exhaustion Intervention: 3 sessions/week of sprint intervals for 6 wks, vigorous intensity; conducted by a subset (*n* = 10) TL: sperm donation before the test (*n* = 34); post PA intervention (*n* = 10)	No significant correlations between TL, fitness or exercise performance Positive correlation between TL and improvements in cardiorespiratory fitness after intervention
Brandao et al. [56]	20 women, premenopausal, obese (20–40 years)	TL: Leukocytes	qPCR	PA: 165 min/week (55 min/session, with 3 sessions/week) for 8 wks PA intensity: 75–90% HR_max_ and increasing multiple RM TL: blood samples pre- and post-PA intervention	↑ Significant increase in rTL with post-PA versus pre-PA ↑ Significant inverse correlation between TL and waist circumference post-PA versus pre-PA
Hernando et al. [123]	149 men and women, healthy, non-smoking (19–67 years) *n* = 96 ultra-trail runners *n* = 53 sedentary controls	TL: Blood; *SOD2 rs4880*: Saliva	qPCR	Endurance athletes: history of ≥ 1 ultra-marathon of ≥ 60 km PA: 107 km ultra-trail race TL: blood samples pre- (1 d before) and post- (1–2 d) competition	↑ Significantly longer TL in older/elderly runners (≥ 40 years) versus age-matched controls Significant association of shorter rTL with *SOD2 rs4880* polymorphism No difference in rTL with previous race training

**Table 3:** The effect of physical activity on telomere biology: *observational studies*

References	Subjects	Telomere analysis: cell type	Measurement method	Physical activity	Main findings
Du et al. [76]	7,813 women (43–70 years) from Nurses’ Health Study (1988 to 1992)	TL: Leukocytes	qPCR	PA: Moderate- or vigorous-intensity, ≥ 3 MET hrs/week Controls: sedentary lifestyle TL: blood collected in 1989 to 1990	↑ Significant increased TL in greater moderate and vigorous intensity activity group (calisthenics or aerobics) versus sedentary group
Savela et al. [77]	204 men (now 76 years) from Helsinki Businessmen Study (1974)	TL: Leukocytes	Southern blot	LTPA: mainly sedentary, low-, moderate-, and high-LTPA, assessed at baseline and at 29 yr follow-up TL: blood samples from a random sub-cohort of survivors at follow-up in 2003	↑ Significantly longer rTL in moderate LTPA group versus low- or high- PA groups Significantly lower proportion of short telomeres in the moderate PA group versus low or high PA group
Denham et al. [63]	123 men, healthy *n* = 67 ultra-marathon runners (44 ± 9 years) *n* = 56 inactive (43 ± 9 years.)	TL: Leukocytes	qPCR	PA: Completion of 2-ultra marathons and average training distance of 40–100 km/week for a minimum of two years Controls: inactive, healthy	↑ Significantly longer TL in ultra-marathon runners versus age-adjusted controls, biological age difference of 16.2 years
Weischer et al. [50]	4,576 men and woman (20–100 years) from Copenhagen City Heart Study (1991 to 94)	TL: Leukocytes	qPCR	PA: ≥ 4 h/week of exercise, inactivity < 4 h/week, and other lifestyle factors, at baseline in 2001–2004 (control) and at 10-yr follow-up TL: blood sampling in 1991 to 94 and at follow-up in 2001 to 03, TL grouped into quartiles	Significant association of shorter TL with physical inactivity (and with several other lifestyle factors) at baseline and at 10-yr follow-up No significant association with TL change at baseline versus 10-yr follow-up
Laine et al. [120]	599 men (72 ± 6 years) *n* = 392 former elite athletes (64 endurance, 221 mixed sport, and 107 power sport athletes) *n* = 207 controls	TL: Leucocytes	qPCR	LTPA assessed for each former elite athlete group over past 3 months, MET-h/week calculated Controls: PA not described	No significant difference in TL between each former elite athlete group and controls No significant differences in TL amongst the different former elite athlete groups
Loprinzi et al. [80]	6,503 men and woman (20–84 years) from NHANES (1999 to 2002)	TL: Leukocytes	qPCR	4 types of PA: moderate, vigorous intensity, transportation PA, and muscle-strengthening, for 30 days MBB index: total number of participated PA types (0 to 4) TL: from blood collected in 2001 to 2003, TL grouped into tertiles	↑ Significant association of longer TL in higher tertile TL group with higher MBB index Dose–response relation between MBB and TL No dose–response relation between TL and muscle-strengthening
Saßenroth et al. [70]	814 men and women (61–82 years) from Berlin Aging Study II (*n* = 452 current PA, *n* = 223 currently inactive)	TL: Leukocytes	qPCR	Current PA: intensive PA, RT, Endurance, or other type of sport, with < 10 years physical inactivity Controls: currently inactive, with ≥ 10 years physical inactivity	↑ Significantly longer rTL with current PA versus currently inactive group ↑ Significantly longer rTL with endurance PA and intensive PA group ↑ Significantly longer rTL with group of PA ≥ 10 years. versus inactive group
Latifovic et al. [71]	477 men and women (20–50 years)	TL: Leukocytes	qPCR	Total amount of PA assessed by IPAQ, grouped into quartiles TL: blood collected from in 2006 to 2008	↑ Significantly longer TL in highest quartile group versus lowest quartile group with vigorous activity
Loprinzi et al. [82]	6,474 men and women (44 ± 0.31 years) from NHANES (1999 to 2002)	TL: Leukocytes	qPCR	9 types of MVPA: ≥ 2000 MVPA MET-min-month, for 30 days (basketball, bicycling, dance, running, stair climbing, swimming, walking, weight lifting) TL: from blood collected in 2001 to 2003	↑ Significant association of longer TL only with running
Sillanpää et al. [81]	386 women, twins, elderly (63–76 years) from the Finnish Twin Study on Aging (FITSA) *n* = 186 monozygotic twins *n* = 200 dizygotic twins	TL: Leukocytes	qPCR	PA level: modified Grimbsy scale (from inactivity to competitive sports weekly) at baseline, 3- and 11-yr follow-up Fitness: maximum distance walked in 6 min, at baseline and at 3-yr follow-up (data missing at 11-yr follow-up) TL: blood collected from 2000 to 2001	↑ Significantly longer TL with higher PA level (and not distance walked) at 3-yr follow-up versus baseline
Denham et al. [59]	122 men and women, healthy (18–55 years) *n* = 61 endurance athletes in cycling, triathlon, or middle-/long-distance *n* = 61 controls	TL: Leukocytes	qPCR	Endurance athletes: competition at national/ international level PA: at least 3 trainings/week, for ≥ 1 yr Controls: recreationally active	↑ Significantly longer TL in endurance athletes (7.1%) and higher TERT expression (twofold) versus age-adjusted controls Resting heart rate was an independent predictor for TL
Denham et al. [40]	84 men and women, healthy *n* = 44 endurance athletes (32 ± 10 years.) in cycling, triathlon, or middle-/long-distance *n* = 40 controls (30 ± 10 years.)	TL: PBMC, Leukocytes	qPCR	Endurance athletes: competition at national/ international level PA: at least 3 trainings/week, for ≥ 1 yr Controls: recreationally active	↑ Significantly longer TL in endurance athletes versus age-adjusted controls, biological age difference of 10.4 years
Dankel et al. [72]	4,881 men and woman (36–85 years) from NHANES study (1999 to 2002) and WATCH paradigm, placed into 6 groups (active or inactive; normal weight or overweight/obese; and normal weight or overweight/obese 10 years ago)	TL: Leukocytes	qPCR	PA: Sports-, exercise-, and recreational-related activities, ≥ 2000 MVPA MET-min-month over past 30 days Control: active, normal weight for ≥ 10 years TL: blood collected from 1989 to 1990	↑ Significant increased odds of longer TL with physical activity in all groups except if overweight/obese for ≥ 10 years versus multi-variate adjusted controls (age, gender, race/ ethnicity, CRP, and change in physical activity level) ↑ Significantly increased odds of shorter TL with inactivity in all groups
Edwards et al. [73]	1,868 men and women (20–49 years) from NHANES (1999 to 2002)	TL: Leukocytes	qPCR	PA: ≥ 1835 MVPA MET-min-month for 30 days CRF: VO_2_ max of ≥ 39 mL/kg/min Sedentary behaviour: ≥ 2 h/day of sitting for 30 days TL: blood collected from 2001 to 2003	↑ Significant association of longest TL with greater physical activity, higher cardiorespiratory fitness and less sedentary behavior Only MVPA was independently associated with longer TL
Ogawa et al. [99]	6,933 men and women (20–84 years) from NHANES (1999 to 2002)	TL: Leukocytes	qPCR	3 groups of moderate PA: < 150 min/week, 150–300 min/week, and ≥ 300 min/week (or ≥ 150 min/week moderate to vigorous PA) for 30 days (household/yard work, transportation PA, moderate and vigorous LTPA) TL: from blood collected in 2001 to 2003	↑ Significant association of longer TL with increment of 1 h/week of moderate PA, vigorous LTPA, or household/yard work ↑ Significant association of longer TL with moderate PA ≥ 300 min/week
Shadyab et al. [74]	1,476 African American and white women, postmenopausal (50–79 years) from Women’s Health Initiative Study (2012–13) with follow-up in the Long Life Study (2012 to 2013)	TL: Leukocytes	Southern blot	PA: total amount, light, MVPA hr/week calculated via hip-worn accelerometer ≥ 10 h/day for 7 days TL: blood collected from 2012 to 2013	↑ Significantly longer TL with ≥ 2.5 h/week MVPA group versus < 2.5 h/week MVPA group
Shadyab et al. [79]	1,476 African American and white women, postmenopausal (50–79 years) from the Women’s Health Initiative Study (1974 to 1978) with follow-up in the Long Life Study (2012 to 2013)	TL: Leukocytes	Southern blot	LTPA: light, moderate, vigorous, with MET-h/week calculated based on total PA, TL: blood collected from 2012 to 2013	↑ Significantly longer TL with higher MET-h/week of LTPA ↑ Significantly longer TL in ≧17 MET-h/week group versus < 1.25 MET-h/week group Significant linear association of moderate to vigorous PA with TL; no association between light PA and TL Associations of TL with PA did not vary by race/ethnicity
Fretts et al. [75]	2,312 American Indians (40 ± 16 years) from Strong Heart Family Study (2001 to 2003)	TL: Leukocytes	qPCR	PA: Steps/day via pedometer, with ≥ 3 days of recorded steps, grouped into quartiles Control: lowest quartile of measured steps/day TL: blood collected from 2001 to 2003	↑ Significant correlation between longer TL and greater number steps/day in all 3 upper quartile groups versus control group
Colon et al. [60]	14 men, healthy *n* = 7 competitive triathletes (35.11 ± 5.86 years) *n* = 7 recreationally active controls (34.14 ± 10.29 years.)	TL: Whole blood	qPCR	Competitive triathletes: competition at the national/ international level Controls: recreationally active	↑ Significant association of longer TL in competitive triathletes versus controls
Aguiar et al. [61]	32 men, heathy *n* = 21 master athletes (51.62 ± 8.19 years) *n* = 11 recreationally active controls (45.41 ± 10.34 years)	TL: Leucocytes	qPCR	Master athletes: competition at the national/international level (in 100 m to marathon distance events) Controls: recreationally active	↑ Significantly longer TL in master athletes versus controls
Stenbäck et al. [65]	700 men and woman (68.9 ± 0.6 years) from City of Oulu Finland health survey follow-up (2013 to 2015)	TL: Whole blood	qPCR	PA: total steps calculated via wrist-worn accelerometer for 2 weeks, and questionnaire of PA intensity (light, moderate, or vigorous), PA history (at 15, 30, 50, and current age), and sedentary time, grouped into quartiles TL: blood collected from 2013 to 2015	↑ Significant positive correlation of longer rTL with higher volume of steps in men, not in women ↑ Significantly longer rTL in the highest quartile moderate PA exercise versus lower three quartiles Significantly longer rTL in women versus men at 69, but no differences at 68 and 70 years of age No association between current rTL and PA in earlier years (age of 15, 30, 50)
Aström et al. [78]	1,035 men (*n* = 453) and women (*n* = 582) elderly, healthy (61 years at baseline) from the Helsinki Birth Cohort Study (2001 to 2004)	TL: Leucocytes	qPCR	PA: SFT (strength, flexibility, and endurance) at baseline in 2001–2004 (control) and at 10-years follow-up TL: blood collected from 2001 to 2004 and from 2011 to 2013	↑ Significant association of longer TL with better physical performance in women at follow-up Shorter TL and greater TL attrition associated with poorer physical performance in women at follow-up
Rosa et al. [95]	60 men, healthy *n* = 31 athletes (18 endurance 53 ± 8 years, 13 sprinter 50 ± 9 years) *n* = 29 controls (12 middle-aged 45.5 ± 10 years., 17 young 22.7 ± 4 years)	TL: Serum ADMA	ELISA	Athletes: ≥ 20 years continuous training, current competition at national and international level in endurance (10,000 m to marathon) and sprint (60 m to 400 m) events Controls: inactive TL control: blood sample from one young male	No difference in rTL between endurance athletes and sprinter athletes ↑ Significantly longer rTL in sprinter athletes versus middle-aged controls
Bastos et al. [96]	53 men, elderly, healthy (66–75 years) *n* = 23 low physical fitness *n* = 7 moderate physical fitness *n* = 23 high physical fitness	TL: T-cells (CD4^+^, CD8^+^ CD28^+^)	MACS, FACS, Flow-FISH	PA: VO_2_ max as per ACSM TL control: 1301 (human T-cell leukemia) cell line	↑ Significant association of longer TL in CD8^+^CD28^+^ cells of the moderate physical fitness group versus cell-line control
Hagman et al. [62]	140 healthy men *n* = 35 young, elite football players (35 ± 0.5 years) *n* = 35 elderly, football players (72 ± 0.5 years) *n* = 35 young, untrained (35 ± 0.6 years) *n* = 35 elderly, untrained (70 ± 0.7 years)	TL & Telomerase: MNC	FISH, qPCR, TRAP assay	PA of young footballers: ≥ 2nd Division in Denmark, ≥ 4 football sessions/week, and ≥ 10 years of regular football training PA of elderly footballers: > 40 years of regular football training and still ≥ 1 football session/week Controls: no regular physical exercise for ≥ 1 yr or no history of participation in sports at a high level earlier in life	↑ Significant increase in TL in the elderly football players group versus elderly untrained control group ↑ Significant Increase in telomerase activity and expression of telomere stabilizing proteins in young football players versus young untrained controls
Jantunen et al. [49]	1,014 men and women, elderly (56–69 years) from Helsinki Birth Cohort Study (2001 to 2004)	TL: Leukocytes	qPCR	LTPA assessed over past 12 months, MET-h/week calculated in 2001 to 2004 (baseline LTPA), grouped into quartiles TL: baseline and 10 yr follow-up rTL: blood collected from 2001 to 2004 and at follow-up from 2011 to 2013	No significant association of TL (at baseline and at follow-up) with LTPA at baseline No significant association of rTL with LTPA at baseline for men Significant inverse relationship in change in rTL with amount of LTPA at baseline in women
Nickels et al. [48]	107 men and women (20 ± 5 years) *n* = 51 swimmers (22 female, 29 male) *n* = 56 controls (29 female, 27 male)	TL: Buccal cells	qPCR	Swimmers: competing at the national/international level Controls: recreationally active, with ≥ 150 min/week moderate PA or ≥ 75 min/week of vigorous PA	↓ Significantly shorter TL in female swimmers versus female controls No significant difference in TL between male swimmers and male controls No correlation in TL between swimming performance, weekly training distance or competing level with TL

Several studies (33/43) found a statistically significant difference in TL (absolute or relative change) with physical activity, based on grouping or stratification of participants’ characteristics, level of fitness, physical activity type, and/or corrections for potential bias. A small number of studies (5/43) reported a significant correlation of TL with level of physical fitness; and an even smaller number (4/43) described a negative correlation between TL and exercise.

## Discussion

A total of 43 studies were selected, and the majority (33 out of 43 studies) highlighted positive effects of exercise on telomere dynamics. Four studies, however, described telomere shortening as a result of physical exercise [47–50]. To further characterize the quality of the studies, the articles were examined and grouped based on their study type of namely RCTs, interventional, and observational studies (Tables 1, 2, 3). In comparison with a systematic review that covered all years up to 2014 but found only one RCT study [46], 37 out of the 43 selected studies in this review were newly-identified articles, including seven new RCTs. A similar yet different systematic review by Arsenis in 2017 that also included studies with influencers such as chronic stress found only 3 RCTs [51].

Further analysis of all 43 of the selected studies showed that the findings are not also completely coherent, which is elaborated in greater detail below.

### RCT Studies

Upon closer examination (Table 1), the RCT studies were inconsistent in their findings, with five of the eight RCTs supporting TL preservation or lengthening with physical activity. The discrepancies amongst the three RCTs may be attributed to differences in the physical characteristics of the participants (e.g. age, obesity, sex-specific, and level of physical activity). Friedenreich et al. [52] conducted an RCT trial on a study population of 212 physically inactive, postmenopausal women. An aerobic exercise intervention at 70–80% heart rate reserve was prescribed for twelve months, and TL was measured and compared against inactive peers. The results showed no significant changes in leukocyte TL. The authors hypothesized that the actual impact of exercise on telomere dynamics depends on various factors of the participants at their baseline, such as body mass index (BMI) or nutrition habits. In another RCT study on a larger population of 439 postmenopausal, overweight women, Mason et al. [53] also showed no significant changes in TL after twelve months of aerobic exercise intervention compared to sedentary controls. Furthermore, weight loss was not associated with an alteration of TL. However, TL was positively associated with maximal oxygen uptake, which is a crucial determinant in setting the upper limit of oxygen uptake for endurance performance [54]. In contrast, some of the selected RCT studies showed increased TL [55–57] or telomerase activity [58] with exercise. One possible explanation for this discrepancy might be attributed to the duration of the exercise intervention of approximately six to twelve months, which could be too short of a timeframe to evoke any significant long-term changes in TL. Thus, cohort studies involving an athlete population could provide some valuable information in this context, since the effects of regular exercise and a high training volume over a longer period of time can be considered.

### Interventional Studies

As shown in Table 2, several of the interventional cohort studies on TL in high-performance athletes describe a positive association of TL with regular and extended participation in physical exercise [59–63]. However, Nickels et al. [48]. reported a significantly shorter TL of 8.1% in young elite swimmers in comparison with their recreationally-active peers, with greater telomere shortening observed amongst female athletes. These conflicting findings might be attributed to differences in exercise intensity, excessive training volume, or even in the type of sport activity itself. Also, the impact of these variables may also be sex-specific. Indeed, some studies suggest sex-specific variations in telomere dynamics [64], and that these differences seem to impact the exercise-induced effects on TL [49, 65]. Of note, this study by Nickels et al. involved a fairly young cohort of athletes (mean age of 20 years); thus, it is unclear whether these findings can be extrapolated to the adult and elderly population. Furthermore, TL is relatively stable until young adulthood, and telomere attrition occurs predominantly at an advanced age [16]. Multiple factors such as age [66], BMI [67], sex [68], volume of training, training modality [57], duration of physical activity, and method of TL analysis [69] can all affect telomere dynamic measurements.

### Observational Studies

Amongst the 27 selected observational studies (Table 3)**,** several cross-sectional studies highlight a positive correlation between exercise and telomere biology [60, 62, 70–77]. The *Helsinki Birth Cohort* was utilized by Ästrom et al. [78] to investigate the correlation between physical performance and TL in the elderly with a mean age of 61 years. Physical performance was assessed using the Senior Fitness Test at the start of the study and at approximately ten years in follow-up. Poorer physical performance correlated with statistically significant telomere shortening after ten years in women [78]. Several other cross-sectional investigations also showed similar statistically significant effects of physical exercise on telomere dynamics [79–81]. Although the results from cross-sectional studies seem promising, such epidemiologic studies are limited by characteristics in their design (e.g. self-reporting of physical activity by questionnaire). Furthermore, evaluating changes in TL from physical activity via a cross-sectional study is limited to measurements that are conducted at a single time point. Thus, in order to assess whether physical exercise counteracts age-related telomere attrition, better consensus amongst observational studies and/or higher-quality evidence from prospective studies is required in order to prove causality.

**Fig. 2:**
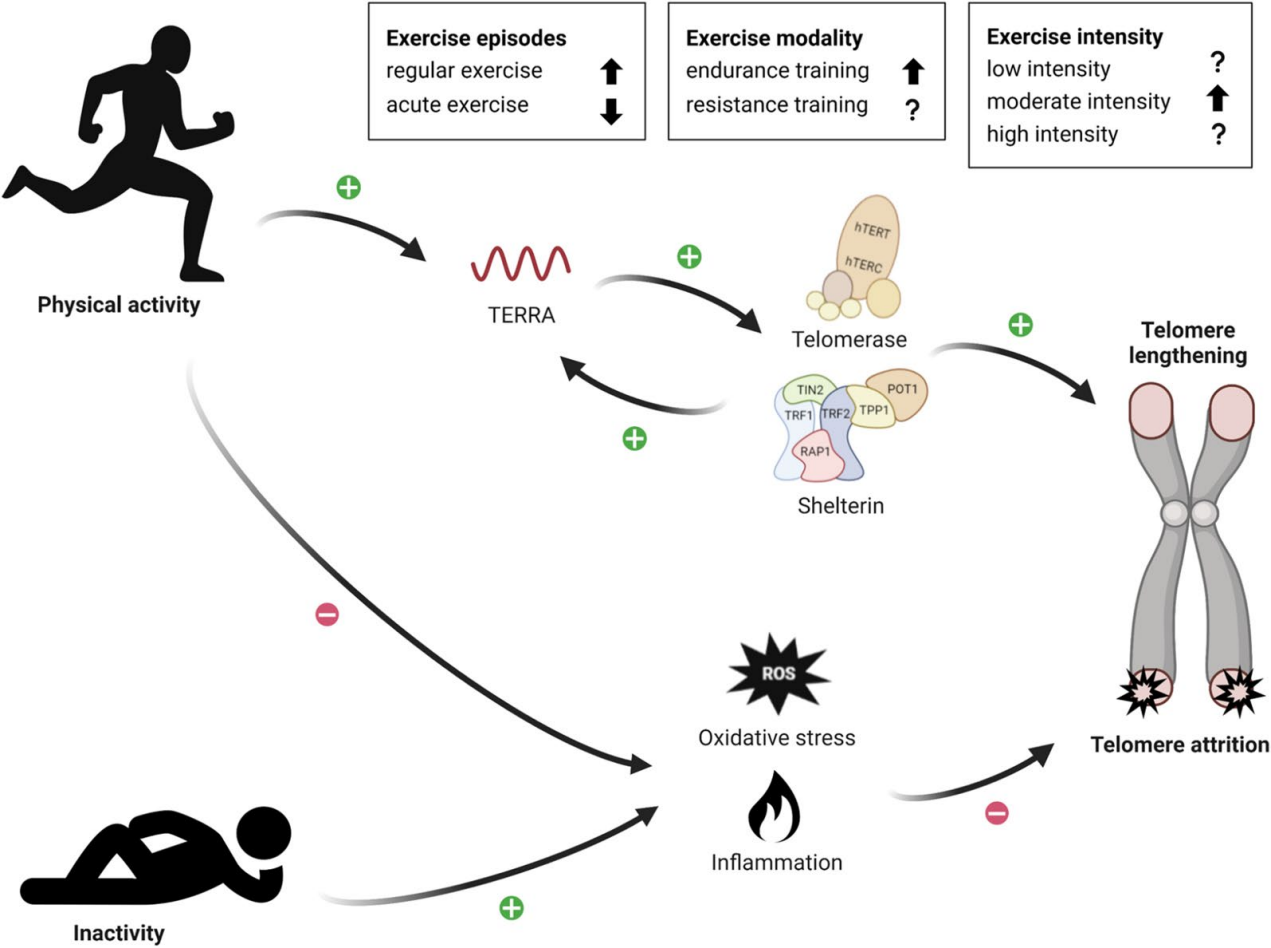
Summary schematic on the potential impact of exercise on telomere length and attrition. Created with Biorender.com

Taken together, a causal relation of physical activity on TL can neither be asserted nor rejected amongst the selected RCT, interventional, and observational studies. Several aspects regarding the type of training modality, and the intensity and duration of the physical activity on telomere dynamics need to be further elucidated, and are further discussed in the following sections. A summary illustration is presented in Fig. 2. Future studies should address influencing factors on telomere biology, along with taking measurements of TL across different time points, which are also described below.

### Effects of Training Modality and Type of Physical Activity on Telomere Biology

Training modality appears to be an important factor to consider regarding its influence on telomere dynamics. Nevertheless, such consideration of different training modalities on telomere biology are scarce. In one RCT study, Werner et al. [57] compared the impact of different exercise modalities (e.g. resistance training or RT, endurance training, and interval training) on TL and telomerase activity. Endurance and interval training protocols increased TL and telomerase activity; however, RT did not register any such differences. Thus, although RT could be integrated into other exercise modalities, RT alone may not be sufficient as a substitute for endurance or interval training to provoke any changes in TL.

With respect to type of exercise, an observational study by Loprinzi et al. on the comparison of different physical activities of various metabolic demands (e.g., weight lifting, basketball, bicycling, running) showed that only running could be positively correlated with preserving TL [82]. This association of TL preservation with running might also help to explain the protective effects of aerobic exercise on the incidence of cardiovascular disease and all-cause mortality [83] and that several chronic diseases are associated with telomere attrition [40, 84]. One possible explanation for the inconsistent findings of RT on TL might be that aerobic exercise (which is not typical in classical RT) is linked to a higher mean and maximum heart rate, which leads to higher vascular shear stress [85]. Since nitric oxide (NO) synthase and telomerase activity are associated with exercise-induced vascular protection [86], the elevated levels of NO released from the vascular wall might contribute to alterations in telomere dynamics. In another study, RT was implemented over six months in an RCT trial, and no clear relationship was shown between RT and TL [87]. In contrast, another RCT study implemented RT for only three months; the results showed an increase of telomerase enzyme activity and other proteins potentially associated with the process of biological aging, namely Sirtuin-1 (SIRT1), SIRT3, and SIRT6 [58]. The seemingly beneficial results of this study might also be attributed to the cohort characteristics of elderly men who performed a more demanding RT protocol in comparison with peers described in other studies. Of note, higher appendicular skeletal muscle index (ASMI) is related to longer telomeres [88]. Since RT aims to increase muscle mass, the preservation of skeletal muscle has also been suggested to help delay telomere attrition and attenuate biological aging.

With respect to endurance training, aerobic exercise emerges as a promising intervention to help maintain or even increase TL. In an RCT study by Puterman et al. [55], aerobic exercise over six months led to significantly longer TL in older adults who were previously inactive and highly-stressed in comparison with sedentary controls. Of note, no increase in telomerase activity was detected, even though such an increase was hypothesized to help stabilize and potentially elongate TL [17, 89]. One reason to account for this increase in TL (in the absence of increased telomerase activity) is that the study cohort consisted of chronically-stressed and inactive individuals, and such characteristics are associated with higher levels of oxidative stress and inflammation [90–92]. Reducing levels of inflammation and oxidative stress with aerobic exercise may have resulted in the reported telomere lengthening [93, 94]. Moreover, endurance exercise was strongly linked to longer telomeres in an athlete study population: ultra-distance runners had 11% longer leukocyte TL in comparison with their peers, which amounts to approximately 16 years of age-related telomere shortening [63].

Overall, endurance exercise is the most investigated training modality and is mainly associated with positive effects on telomere biology. Moreover, some types of exercise may have greater benefit than others. In one observational study, a comparison between master sprinters and endurance runners revealed a better profile of aging biomarkers in sprinters, including a trend for longer TL. Endurance athletes had a better NO profile, which is considered a marker of endothelial function, whereas sprinters possessed a significantly better redox balance and cytokine profile [95]. Future studies would need to clarify whether running itself or the intensity associated with running affects telomere dynamics.

### Effects of Exercise Intensity on Telomere Biology

Training intensity is a crucial factor not only in exercise training programs but also in the setting of telomere biology and aging [77]. However, the selected studies present inconsistent findings on whether moderate or highly intense exercise has a beneficial impact on telomere dynamics. Du et al. examined participants enrolled in the large-scale Nurses’ Health Study and showed that moderate exercise (defined as energy exposure of 3 METs or more) is already sufficient to preserve leukocyte TL in women [76]. These findings are in keeping with the results of Savela et al. [77]. Namely, the relationship between physical activity level (in terms of intensity and frequency) and TL was described as an inverted U-shaped curve, indicating that a moderate level of exercise shows a beneficial effect on TL; in contrast, low- and high-level of physical activity were associated with shorter telomeres. Furthermore, a moderate level of physical activity likely counteracts age-dependent immunosenescence and prevents telomere shortening in specific T-cell populations. Namely, in a study by Bastos et al. [96]., low- and even high-levels of physical activity did not significantly affect immunosenescence in CD8^+^CD28^+^ cells in comparison with a cell-line control. Taken together, these findings suggest there are possible positive effects of physical exercise on the immune system in older adulthood and that the most beneficial exercise intensity level in this context is exercise at moderate intensity.

In comparison with other levels of exercise intensity, Colon et al. [60] showed that TL was better preserved through high-intensity training in comparison with exercise at moderate intensity. This study was conducted with competitive triathlon athletes at a high level of fitness as the investigated cohort. As such, amongst the triathletes, the applied training protocol might not have reached the required intensity associated with detrimental effects to TL as seen in other studies involving non-athletes. In a study by Denham et al. [63], ultra-endurance athletes possessed 11% longer telomeres (an approximate increase of 324–648 bp) in comparison with healthy inactive controls, which corresponds to a difference of 16.2 years in biological age. Recent studies [97, 98] also underline the importance of exercise intensity on telomere biology, and are in keeping with the results from Gagnon et al. [98]. In participants of a multi-day canoeing expedition (travelling six hours a day for 2 weeks) at low-to- moderate intensity, TL remained unchanged in comparison with age-matched controls; however, there was a significant improvement in their metabolic and oxidative profiles. The increased enzymatic antioxidative activity was even comparable to the redox-balancing benefits induced by a training duration of several months. Nevertheless, the exercise stimulus might have been insufficient to influence TL, since the physical activity was at a low-to-moderate intensity [98]. Other studies [70, 71, 99] reported a positive dose–response relationship between relative telomere length (rTL) and physical activity. A significant linear trend of increasing rTL with vigorous physical activity emerged, but moderate physical activity was not necessarily associated with any changes in TL.

In summary, the selected studies appear contradictory on what exercise intensity has the greatest influence on telomere dynamics. Also, the level of physical activity (e.g. low, moderate, or high/vigorous) is not always clear or described in greater detail in terms of its parameters (e.g. intensity, duration, and frequency) to allow comparison. Since different types of physical activity generate different metabolic demands [100], further research is needed to determine whether an optimal level of exercise parameters can be achieved to slow down the biological process of aging.

### The Impact of Acute Exercise Episodes on Telomere Biology

Previous research suggests that acute exposure to strenuous exercise induces an increase in oxidative stress [101, 102]. DNA damage from oxidative stress has been shown to occur even after a few hours of exposure to high-intensity aerobic exercise [103, 104]. Since oxidative stress is also strongly associated with telomere attrition [105] by inhibiting telomerase activity [106], the effects of acute bouts of exercise on TL require closer examination. Borghini et al. [47] reported a significantly reduced rTL in athletes during and after completing an ultra-long running distance endurance race (330 km) as a result of oxidative DNA damage. However, TL was better preserved amongst athletes in comparison with sedentary controls; thus, these findings imply that regular endurance exercise still has beneficial effects on TL over a longer period of time in comparison with inactivity. Antioxidant mechanisms might be sufficient in handling an increase in reactive oxygen species (ROS) during regular endurance exercise due to adaption over time; however, such regulation mechanisms of ROS might be overwhelmed by an elevation of oxidative stress from an acute episode of high-intensity exercise.

In contrast, Laye et al. [107] showed no statistically significant changes in TL after exposure to extreme physiological stress of completing seven marathons in seven days. Of note, TL was analyzed in peripheral blood mononuclear cells or skeletal muscle biopsies. As previously mentioned, this non-change might be attributed to the above-average physical fitness level of the athletes. These high-performance, long-distance athletes might be less affected by ROS, which might correlate with a milder effect of oxidative stress on telomere biology. As such, these findings may not translate to the normal non-athlete population. Moreover, telomerase activity, which is described as a more accurate marker of muscle turnover than mean TL [108], could not be detected in the skeletal muscle biopsies.

Although some evidence suggests an acute bout of exercise has an immediate and detrimental effect on TL, prolonged exposure to endurance exercise seems to attenuate TL attrition, contribute to TL maintenance, or even promote TL lengthening via milder ROS effects.

### The Impact of Inactivity on Telomere Biology

Inactivity and sedentary behavior are reported to influence telomere biology [65], with strong association with worse health outcomes and higher cardiovascular disease risk [109, 110]. Especially in Western countries, while formal participation in exercise has increased, a sedentary lifestyle has become more prevalent [111]. Along with cardiovascular disease, there is an elevated risk for other chronic diseases associated with a sedentary lifestyle (e.g. metabolic syndrome, type 2 diabetes) that is independent of the time spent on exercising [111–114]. Sedentary behavior is also linked to decreased muscle mass and low muscle strength [115]. Furthermore, prolonged sitting is discussed to elevate inflammation and oxidative stress levels [116, 117], subsequently contributing to telomere attrition. In an RCT study involving elderly, sedentary, and overweight subjects, Sjögren et al. [118] reported significant telomere lengthening by reducing sitting time; in contrast, increased time spent on exercising was associated with telomere shortening. These results indicate that reducing the time spent sitting in an elderly at-risk population might be of greater importance for TL maintenance than participation in actual exercise. Fretts et al. [75] also underline the importance of movement on a regular basis; study participants who accumulated more steps per day had significantly longer TL and vice versa. Despite the cross-sectional study design with a single time point of data collection, this study stands out from similar investigations because of its objective measurement of activity where a pedometer was used to count the daily steps instead of self-reported questionnaires. In comparison, an investigation of Edwards et al. [73] showed only a significant association with TL in participants who engaged in moderate-to-vigorous physical activity (for METs ≥ 3); and no correlation between the number of hours of sedentary behavior and TL was detected.

In summary, the reduction of sedentary time appears to have a positive impact on TL preservation; and physical activity may not alone be able to attenuate the adverse effects of sedentary behavior. The findings from the selected studies seem slightly divergent, on whether sedentary behavior or physical activity itself substantially impacts telomere dynamics. Nevertheless, higher physical activity levels and reduced sitting time are both strongly associated with TL preservation and attenuating the aging process at a molecular level.

### Effects of Previous Exercise History on Telomere Biology

Although numerous studies promote the beneficial effects of physical exercise on TL, further clarification is necessary on the required timeframe of the training stimulus. For instance, former athletes are associated with a healthier metabolic profile and a lower prevalence of developing cardiovascular risk factors [119–121]. Having a history of life-long training is even associated with greater longevity in comparison with the general population [122]. Rosa et al. showed that life-long training volume is proportional to TL [95], which is in parallel to the findings by Hernando et al. [123]. Even though extreme endurance exercise has been associated with elevated levels of oxidative stress, TL was better preserved in ultra-endurance athletes than in their age-matched peers [123], particularly evident in elderly athletes who had been training on a regular basis over many years. In comparison, there was a lack of significant differences in TL between young athletes and their age-matched inactive controls, most likely due to the fewer number of years engaged in regular training. These findings are consistent with a study by Borghini et al. [47]. Namely, TL was also better preserved in older endurance athletes in comparison with age-matched inactive controls, and no changes in TL were reported amongst younger endurance athletes.

In contrast to an athlete population, a combined exercise intervention of strength and aerobic training showed significant telomere lengthening in leukocytes as early as eight weeks in a population of obese, premenopausal women [56]. These results suggest that perhaps a history of physical activity plays less of an influential role than an individual’s health status and physical fitness level at baseline. In fact, Laine et al. [124] showed that former elite-class male athletes who restarted training did not display any significant differences in TL later in life compared to their age-matched active controls. The history of vigorous training in these participants was limited in their young adulthood, which is a stage in life when exercise training might only exhibit a minor influence on telomere biology [125]. In addition, engagement in regular exercise supports telomere maintenance in the elderly, regardless of physical activity in early adulthood [65, 70]. Nevertheless, Saßenroth et al. showed that longer periods of physical activity (over ten years) are associated with longer rTL in comparison with inactive controls [70]. However, it is unclear as to when the period of activity (or inactivity) had occurred. Therefore, it appears that it is never too late to start exercising and benefit from its positive effects on health and telomeres. Exercise seems to have a stronger impact on TL preservation in later years, since TL attrition itself manifests to a greater degree later in life, especially after 70 years of age, with associated exacerbated senescence [126].

### Effects of Exercise on TL at Different Ages?

For in vitro studies, there are convincing data on how TL is associated with the replicative capacity of a cell. However, due to inter-individual differences attributed to endogenous (e.g. ethnicity, gender, genetic factors, BMI) and exogenous factors (e.g. lifestyle choices and environmental stressors), the process of biological aging and its effect on TL is much harder to unravel in vivo [14]. Furthermore, assessing the impact of exercise on TL presents further challenges, since differences in training parameters or the type of exercise may show different effects.

Amongst the selected studies, only a very few addressed the effects of exercise on TL by comparing different age groups [62, 95, 123, 127]. Furthermore, the majority of the selected studies involved participants over 60 years of age [49, 52, 53, 55, 58, 62, 65, 70, 77, 81, 87, 96, 118]. For RCTs, two out of the eight studies involved subjects in their late 40 s and 50 s, and the remaining focused on subjects over 60 years of age (Table 1). Thus, along with the lack of data amongst younger subjects, further data comparing the effects of exercise on TL at different age groups is required.

The impact of exercise on TL might demonstrate a more pronounced beneficial effect from the mid-40 s and onwards, as several factors that accelerate telomere attrition are also associated with aging. One of these influencing factors might be the change in body composition from a decrease in lean mass to an increase in adipose tissue with age. [128, 129]. Physical exercise contributes both in the maintenance of skeletal muscle mass and in reducing body fat. As increased body fat composition is linked to telomere attrition, the benefits of physical exercise are twofold. [130]. This finding is in keeping with the observational study by Aguiar et al. [61] that highlighted an inverse correlation between body fat and TL in middle-aged master athletes compared to untrained age-matched controls.

### Possible Molecular Explanations for the Observed Discrepancies in TL with Physical Activity

Changes in TL might be a more dynamic process than previously assumed. Weischer et al. [50] reported significant findings for both telomere shortening and elongation, which were found in 56% and 44% of all the participants respectively (*n* = 4,576) at 10-year follow. In another study, even within two years of follow-up, there was a significant decrease in TL [65]. Indeed, how exercise modulates telomere dynamics is not yet fully understood. Several studies provide possible explanations, highlighting crucial influential factors and molecular processes [61, 127, 131]. Possible explanations that could account for such discrepancies in TL are further described below.

### Tissue-Specific and Cell-Specific Differences in Telomere Biology

As part of the Genotype Tissue Expression (GTEx) project on post-mortem tissues, TL has been shown to be negatively correlated with age in the majority of the > 20 different tissues examined [132]. This inverse association of TL with age was most remarkable amongst tissues from the aorta, stomach, whole blood, and kidney. However, TL from muscle was found to be neither positively nor negatively correlated with age. Furthermore, amongst different samples of tissue from the same organ and from the same individual, TL can vary by a factor of sixfold or more [133]. Whole blood may be an attractive model-candidate for TL analysis, based on its accessibility and processibility: however, it is also a tissue that is prone to telomere biology disorders (TBDs) which are characterized by loss of function mutation in telomere maintenance genes resulting in shorter TL.

Almost all of the selected studies analyzed TL using leukocytes and/or whole blood. However, telomere shortening and the impact of exercise on telomere dynamics might be cell-type specific [134, 135]. Also, TL varies across different somatic tissues in proportion to their replicative activity [136]. Therefore, the results of studies that analyze TL from one cell type cannot necessarily be generalized to other cell types. Changes in TL result not only from the frequent replication of somatic cells, but also from exposure to environmental toxins; and both are associated with a diminished function of post-mitotic cells [137]. Skeletal muscle, for instance, contains mainly post-mitotic myonuclei, which would suggest that the TL remains relatively constant and unchanged due to the small number of cells undergoing replication and turnover during a lifespan [138]. Magi et al. [139] demonstrate the differences in TL across different cell types, where TL was longest in muscle cells and shortest in leukocytes. However, TL was strongly correlated between these tissues when differences in their replicative activity were considered. Namely, telomere attrition rates were similar in highly proliferative blood cells compared to minimally proliferative muscle cells. As such, one proposal has been to normalize TL in leukocytes against TL in a post-mitotic tissue like skeletal muscle or fat [42]. However, in a study by Hiam et al. [140] that investigated the effect of aerobic capacity on TL in leukocytes and muscle across a broad age range of 18 to 87 years, there was no association of TL in skeletal muscle with aging and longer TL was not observed in either leukocytes or skeletal muscle. One possible explanation for this discrepancy is that TL varies amongst different lymphocyte subpopulations [141, 142]. As such, there is also the possibility that the observed TL changes could be an artifact caused by a redistribution of leukocyte subpopulations. Moreover, telomeres of leukocytes shorten by only about 33 base pairs per year [143], which is hard to detect with standard telomere measurement methods. TL also oscillates over time in whole blood samples due to shifts in cell populations [144].

Since telomeres are susceptible to oxidative stress [145], the antioxidant capacity of different cells (especially immune cells) could lead to differences in TL, especially in a study cohort of endurance athletes. In this context, Ludlow et al. [135] suggest that the protective effects of chronic exercise regarding age-related telomere shortening are cell-specific. This finding is also congruent with the study by Denham et al. [134], whereby TL derived from whole blood leukocytes was longer (by 6.1% on average) amongst endurance athletes in comparison with inactive controls. However, no significant difference in TL derived from peripheral blood mononuclear cells was observed. Granulocytes might play an important role in investigating the effects of exercise on telomere dynamics, since these cells represent up to 75% of the leukocyte population. As glycolysis is the predominant metabolic pathway of granulocytes [146], granulocytes might be more influenced by intense exercise training. As such, the effects of exercise on TL in leukocytes may be more reflective of its effects on TL in granulocytes. Finally, in comparison with other cell types. Leukocytes have a relatively short lifespan of up to 3 days. Taken together, differences in TL across different tissue types and heterogenicity in subpopulation cell-types need to be carefully considered in TL analysis.

### Effects of Oxidative Stress on Telomere Biology

Increased levels of oxidative stress due to free radicals and/or a decrease of antioxidants are presumed causes of telomere attrition and aging [147]. With regular physical exercise, pro-oxidant levels are reduced and antioxidant defense mechanisms are enhanced, resulting in an improved oxidative balance [94, 148]. Furthermore, the elevation of oxygen uptake with intense exercise induces an increase of superoxide radicals and other ROS, which leads to elevated levels of oxidative stress. However, exercise on a regular basis provokes similar adaptations seen with acute bouts of training by upregulating antioxidative enzymes, thereby improving the redox balance [149]. These changes might further reduce oxidative DNA damage, and thus diminish age-dependent telomere shortening [139, 150, 151]. The findings by Aguiar et al. [61] are in concordance with previous studies, showing that sprinter athletes had a better oxidative profile and longer telomeres compared to their age-matched controls. Furthermore, body fat was inversely correlated with both TL and markers of oxidative stress, further highlighting the negative effects of adiposity in aging.

### Proteins and Molecular Pathways that Influence Telomere Biology

Capping the ends of chromosomes, telomeres, by definition, include both the repeating nucleotide sequences (e.g. G-strand or repeating 5'-TTAGGG-3' sequences, C-strand or repeating 3'-AATCCC-5' sequences and G-rich overhang or G-overhang) and their associated proteins. Telomerase, a ribonucleoprotein, elongates telomeres through its own intrinsic RNA sequence and reverse transcriptase enzyme. As such, telomere maintenance involves factors that influence telomerase activity through tertiary telomeric structures (e.g., T-loop formation, D-loop formation, telomeric repeat-containing RNA or TERRA) and through specialized proteins (e.g., shelterin and the CST complex) [152].

In brief, shelterin is a complex of 6 proteins (TRF1, TRF2, POT1, RAP1, TIN2, and TPP1) that bind to telomeric DNA, with each shelterin protein having its own unique function in telomere maintenance. For example, TRF1 helps to upregulate TERRA transcription [153]; TRF2 is involved in the formation of T-loop structure which acts as a physical obstacle against aberrant activation of DNA-damage repair (DDR) mechanisms; and RAP1 has been shown to help promote epigenetic-silencing of genes proximal to telomeres, a process known as the telomere position effect (TPE) [154]. When gene silencing occurs over long-distances of DNA via long telomeres looping back in the chromatin, the phenomenon is referred to as TPE-over long distances or TPE-OLD. The expression of *hTERT* is thought to be regulated by a TPE-OLD [155, 156]. Both TTP1 and POT1 are involved in regulating telomerase by promoting processivity. In contrast, the interaction of telomerase with the CST complex (which consists of three proteins of CTC1, STN1, and TEN1 or CST in humans) is thought to inhibit telomerase activity, thereby preventing aberrant telomere extension [157].

Accelerated cell aging is often explained by a lack of telomerase activity [158, 159]. Exercise has been shown to increase telomerase activity measured by an increased expression of *hTERT*, with TERT as the crucial catalytic subunit of telomerase [160]. Consistent with these findings, Cluckey et al. [127] reported increased *hTERT* expression after an acute bout of high-intensity exercise, and this increase attenuated with age. Denham et al. [161] examined 15 retrieved studies on mammals (human and rodent) and found that a single bout of exercise or long-term exercise upregulated *TERT* and telomerase activity in non-cancerous cells. In addition, elderly participants showed a significant upregulation of the shelterin protein TRF2, which is considered a negative regulator of telomerase activity [162], possibly through regulation of *hTERT* expression by TPE-OLD.

Diman et al. [131] identified nuclear respiratory factor (NRF1), the dimeric form of which is a transcription factor involved in cellular growth and metabolism, as a crucial regulator of telomere transcription in vitro. With upstream regulation of NRF1 by adenosine 5’-monophosphate (AMP)-activated protein kinase (AMPK), pharmacological activation of AMPK in cancer cell lines led to an upregulation of TERRA, thereby suggesting a link between cellular fitness and telomere metabolism. By analyzing skeletal muscle biopsies, increased levels of TERRA (which is associated with AMPK activation) were found with endurance exercise of 45 min of cycling. Taken together, these data suggest that the AMPK pathway regulates telomere transcription. Since most telomeres from muscle cells are likely covered with TERRA, it is hypothesized that exercise enables renewing of TERRA pools, subsequently preserving TL [131]. In addition, exercise-induced elevation of blood lactate might contribute to telomere protection by increasing NRF1 and AMPK-mediated expression of TERRA [131].

Overall, telomere biology is a highly complex process, and recent studies support that there are differences in the regulation of TL and telomerase in different tissue- and cell-types. Furthermore, oxidation levels seem to play a crucial role in telomere dynamics during training, both in the acute and chronic setting. Therefore, it remains a challenge to determine which molecular pathways influence telomere biology through physical exercise.

### Methods of Detecting TL and their Limitations

There are a number of different techniques in TL analysis [163]. By far, the most commonly used method in TL analysis is qPCR. This technique involves high throughput amplification of a small amount of telomeric DNA (T) that is compared against a single copy gene (S) to generate T/S ratio. As such, this ratio provides a relative TL in a sample. However, with qPCR, the amount of short and long telomeres, and differences in TL between individual chromosomes cannot be determined. The shortest of telomeres segments propagate DDR and other downstream events like cellular senescence. Therefore, a number of techniques aim to quantify short telomere segments. Terminal Restriction Fragment (TRF) determines the average TL via Southern Blot analysis. However, larger amounts of DNA are required (3 µg) and the detection limit is restricted to smaller telomere fragments of 2 kb. The Telomere Shortest Length Assay (TeSLA) [163] is a technique that involves a small amount of DNA (1 µg) and can detect any of the telomeric ends of chromosomes from < 1 kb to 18 kb, along with average TL. However, TeSLA is limited to low throughput, and quantification of longer telomeres in mice can be problematic. Prior to TeSLA, similar methods of Single Telomere Length Analysis (STELA) [164] and Universal STELA (U-STELA) [165] were developed to detect TL using a combination of ligation, PCR and Southern Blot techniques. Both STELA and U-STELA are also low throughput but require greater amounts of telomeric DNA in comparison with TeSLA. In contrast, Quantitative Fluorescence In Situ Hybridization (Q-FISH) labels telomeric ends of chromosomes for visualization under fluorescence microscopy. Flow-FISH also labels telomeres with fluorescent probes; however this method of detection involves fluorescence activated cell sorting (FACS) analysis. Q-FISH techniques have been instrumental in showing that the shortest of telomeric DNA segments, not the average TL, are crucial for cell viability and chromosomal stability in mice [166]. However, these methods are limited by their hybridizing fluorescent probe or peptide nucleic acid (PNA) probe, which consists of telomeric repeats of CCCTAAA. As such, PNA probes can potentially generate false positive results by binding to other regions of DNA with telomeric repeats located away from chromosome ends.

## Conclusions

This systematic review summarizes and supports the growing body of evidence that physical activity has an impact on telomere attrition and thus on the aging process. While the majority of the included studies highlight positive effects of physical activity on telomere dynamics, there lacks a consensus on the most beneficial exercise type and training modality (intensity, duration and frequency). Furthermore, inactivity is a major risk factor for cardiovascular disease and several other chronic disease conditions, independent of exercise. Notably, the amount of reduction in sedentary behavior has a profound and positive effect on preserving and/or increasing TL. With respect to history of previous exercise, current level of physical fitness appears to have a more important beneficial role than previous exercise on TL. In fact, amongst athletes, a history of physical activity during youth does not appear to play a protective role in preserving or increasing TL. Nevertheless, there is strong evidence that, lifelong elite- or master-athletes will have increased TL in comparison with inactive controls. Although the majority of the studies underscore the beneficial role of physical activity on telomere dynamics and aging, the molecular events in TL preservation and/or elongation remain poorly understood. Along with further understanding of telomere biology and potential deleterious events at the molecular level (e.g. oxidative stress), tissue- and cell-type differences in their analyses of TL and telomerase need to also be considered. Future studies should provide more detailed information on the physical fitness level of the participants, as well as characteristics of the exercise training modality, for standardization and comparison, in order to draw more definitive conclusions.

## Data Availability

Data sharing is not applicable to this article as no datasets were generated during the current study.

## Abbreviations

ACSM: American College of Sports Medicine
ADMA: Asymmetric dimethylarginine
AET: Aerobic endurance training
AMPK: AMP-activated protein kinase
CRF: Cardiorespiratory fitness
CRP: C-reactive protein
CST: CTC1-STN1-TEN1
ddPCR: Droplet digital polymerase chain reaction
ELISA: Enzyme linked immunosorbent assay
FACS: Fluorescence activated cell sorting
FISH: Flow-fluorescence in situ hybridization
GTEx: Genotype tissue expression
IPAQ: International Physical Activity Questionnaire
hTERT: Human telomerase reverse transcriptase
HR: Heart rate
LTL: Leucocyte telomere length
LTPA: Leisure time physical activity
MET: Metabolic equivalent of task
MET-h/w: Metabolic equivalent of task-hours per week
MVPA: Moderate to vigorous physical activity
MNC: Mononuclear cells
NHANES: National Health and Nutrition Examination Survey
NRF1: Nuclear respiratory factor 1
qPCR: (Real time) quantitative polymerase chain reaction
qRT-PCR: Quantitative reverse transcription polymerase chain reaction
PA: Physical activity
PBMC: Peripheral blood mononuclear cells
PNA: Peptide nucleic acid
POT: Protection of telomeres
PWV: Pulse wave velocity
qPCR: Quantitative polymerase chain reaction
qRT-PCR: Quantitative reverse transcription polymerase chain reaction
RAP1: Repressor/activator protein 1
RM: Repetitions maximum
rTL: Relative telomere length
RT: Resistance training
SFT: Senior fitness test
TBD: Telomere biology disorder
TIN2: TRF1- and TRF2-interacting nuclear protein 2
TL: Telomere length
TERRA: Telomeric repeat-containing RNA
TPE-OLD: Telomere position effects-over long distances
TPP1: Tripeptidyl-peptidase 1
TRF: Terminal restriction fragment
TRF1: Telomeric repeat-binding factor 1
TRF2: Telomeric repeat-binding factor 2
TRAP: Telomerase repeat amplification protocol
VO_2_max: Maximal oxygen consumption
WATCH: Weight, activity, and time contributes to health
